# Restriction in lateral bending range of motion, lumbar lordosis, and hamstring flexibility predicts the development of low back pain: a systematic review of prospective cohort studies

**DOI:** 10.1186/s12891-017-1534-0

**Published:** 2017-05-05

**Authors:** Sean G Sadler, Martin J Spink, Alan Ho, Xanne Janse De Jonge, Vivienne H Chuter

**Affiliations:** 1 Discipline of Podiatry, University of Newcastle, Ourimbah, Australia; 2 School of Psychology, University of Newcastle, Ourimbah, Australia; 3 School of Environmental and Life Sciences, University of Newcastle, Ourimbah, Australia

**Keywords:** Low back pain, Systematic review, Risk factors, Prospective cohort studies, Meta-analysis

## Abstract

**Background:**

Low back pain (LBP) is an increasingly common condition worldwide with significant costs associated with its management. Identification of musculoskeletal risk factors that can be treated clinically before the development of LBP could reduce costs and improve the quality of life of individuals. Therefore the aim was to systematically review prospective cohort studies investigating lower back and / or lower limb musculoskeletal risk factors in the development of LBP.

**Methods:**

MEDLINE, EMBASE, AMED, CINAHL, SPORTDiscus, and the Cochrane Library were searched from inception to February 2016. No age, gender or occupational restrictions of participants were applied. Articles had to be published in English and have a 12 month follow-up period. Musculoskeletal risk factors were defined as any osseous, ligamentous, or muscular structure that was quantifiably measured at baseline. Studies were excluded if participants were pregnant, diagnosed with cancer, or had previous low back surgery. Two authors independently reviewed and selected relevant articles. Methodological quality was evaluated independently by two reviewers using a generic tool for observational studies.

**Results:**

Twelve articles which evaluated musculoskeletal risk factors for the development of low back pain in 5459 participants were included. Individual meta-analyses were conducted based on risk factors common between studies. Meta-analysis revealed that reduced lateral flexion range of motion (OR = 0.41, 95% CI 0.24-0.73, *p* = 0.002), limited lumbar lordosis (OR = 0.73, 95% CI 0.55-0.98, *p* = 0.034), and restricted hamstring range of motion (OR = 0.96, 95% CI 0.94-0.98, *p* = 0.001) were significantly associated with the development of low back pain. Meta-analyses on lumbar extension range of motion, quadriceps flexibility, fingertip to floor distance, lumbar flexion range of motion, back muscle strength, back muscle endurance, abdominal strength, erector spinae cross sectional area, and quadratus lumborum cross sectional area showed non-significant results.

**Conclusion:**

In summary, we found that a restriction in lateral flexion and hamstring range of motion as well as limited lumbar lordosis were associated with an increased risk of developing LBP. Future research should aim to measure additional lower limb musculoskeletal risk factors, have follow up periods of 6-12 months, adopt a standardised definition of LBP, and only include participants who have no history of LBP.

**Electronic supplementary material:**

The online version of this article (doi:10.1186/s12891-017-1534-0) contains supplementary material, which is available to authorized users.

## Background

Low back pain (LBP) is an increasingly common condition worldwide, particularly in low and middle income countries, which has a significant negative impact on the quality of life of sufferers [1]. The growing impact of LBP globally is evidenced by recent research indicating that LBP is now among the top ten causes of years lived with disability [2]. Lifetime prevalence of LBP is reported to be as high as 84% with approximately 23% suffering from chronic pain [3], although this is highly variable and depends on the specific population investigated. The economic implications of early retirement and lost productivity, associated with LBP, are alarming with costs to individuals and governments continuing to increase [1].

The aetiology of LBP is multifactorial with previous LBP, frequent bending and twisting, prolonged static postures, anxiety, depression, and somatisation all having been linked to the development of the condition [4, 5]. A number of possible musculoskeletal risk factors have also been implicated in the development of LBP and verification of these may offer a potential mechanism by which LBP can be effectively treated. Accurate identification of musculoskeletal risk factors may also offer a mechanism by which occurrence of LBP can be prevented and the associated socioeconomic burden of the condition reduced.

Dysfunction of muscles of the lumbopelvic-hip complex (core muscles) has been demonstrated to increase spinal loading and reduce spinal stability with altered core muscle recruitment patterns a hallmark of LBP, particularly in a chronic form [6]. Similarly, abnormal lower limb function is proposed to reduce absorption of impact force and affect spinal loading with dysfunction both distally and proximally in the lower limb suggested to contribute to the development of LBP. For example, excessive foot pronation [7–9] and tight hamstrings [10, 11] have been associated with an increased risk of developing LBP. Excessive foot pronation can lead to an internally rotated tibial and femoral position which may encourage an anterior pelvic tilt [12, 13]. The altered pelvis position is thought to increase the strain on pelvic muscles, such as the piriformis, which may cause compression of the sciatic nerve [7, 14]. Additionally, the altered pelvic position is proposed to put strain on intervertebral discs, increasing pain [15, 16]. Tight hamstring muscles may reduce the lumbar lordosis, potentially decreasing the absorption of force, and increasing the possibility of developing LBP [17].

There have been few systematic evaluations of musculoskeletal risk factors for LBP with the majority of work focussing on prospective articles investigating other risk factors such as psychosocial or work related physical activity in the adult population [18–23]. Of existing data in adults, that have specifically investigated musculoskeletal risk factors, the authors found no relationship between trunk muscle endurance or strength, or mobility of the lumbar spine, and the risk of developing LBP [18]. A systematic review in adolescents and children found that there was limited evidence to support a range of musculoskeletal risk factors for the development of LBP [24]. Individual risk factors were demonstrated to be significant in single studies, however, differences in definitions of LBP and measurement techniques between studies prevented meta-analyses. Further to this, the differences in study populations and variable follow-up times make it difficult to draw definitive conclusions. Therefore a systematic review that collectively evaluates the contribution of lower back and lower limb musculoskeletal risk factors in the development of LBP in all age groups, over the short to medium term is required.

This systematic review aims to evaluate prospective cohort studies that have investigated lower back and/or lower limb musculoskeletal risk factors in the development of LBP over 12 months. Secondary aims include the identification of the type and duration of LBP that participants develop. Study findings will be evaluated by meta-analysis where appropriate.

## Methods

### Search strategy

An electronic database search of MEDLINE, EMBASE, CINAHL, SPORTDiscus, AMED and The Cochrane Library was conducted from inception to February 2016. Search terms were adapted for each of the databases (Additional file 1). The PRISMA statement was used to structure this systematic review.

### Eligibility criteria

Only English language prospective cohort studies investigating musculoskeletal risk factors with a 12 month follow-up were included. The length of follow-up was limited to 12 months due to the possibility of other extraneous variables influencing the development of LBP. Musculoskeletal risk factors were defined as any osseous, ligamentous, or muscular structure that was quantifiably measured at baseline. Studies were excluded if participants were pregnant, diagnosed with cancer, or had previous low back surgery. Studies that investigated the development of injuries, with no separate data for those who developed LBP, were also excluded. Studies could report LBP by any means.

### Study selection

One reviewer conducted the electronic searches (SS). Titles and abstracts were independently assessed by two reviewers (SS and MS). No disagreements occurred while screening for inclusion therefore no arbitration by third reviewer (VC) was needed. The reference lists of included studies, clinical guidelines, and recently published systematic reviews were also searched for potentially eligible studies. Data extraction was conducted by one reviewer (SS) using a standardised data extraction form and cross-checked by a second reviewer (AH).

### Quality assessment

Methodological assessment was performed independently by two reviewers (SS and JA) using a generic tool for observational studies developed by Weightman et al. [25]. Included studies were awarded a ‘yes’, ‘no’, or ‘can’t tell’ for each criterion. No disagreement occurred while assessing the quality of included studies.

### Statistical analysis

Effect sizes were either obtained from studies if reported or calculated appropriately from proportions or means and standard deviations using Hasselblad and Hedges methods [26]. There was insufficient information in each study to allow more elaborate modelling that might account for correlations between measures. For all meta-analyses performed, random effects models with DerSimonian and Laird weights were used due to the varying study designs and populations [27]. The I^2^ statistic was used to assess the level of heterogeneity within each of the meta-analyses. In instances where a risk factor was common between two or more studies, it was combined in a meta-analysis [27]. Statistical analysis to assess the risk of publication bias was not used as fewer than 10 studies were included in the meta-analysis and, in these instances, test power has been reported to be too low to distinguish chance from actual asymmetry [28]. Software packages Microsoft Excel 2016 and STATA 12 were used for statistical analyses.

## Results

### Study identification

**Fig. 1:**
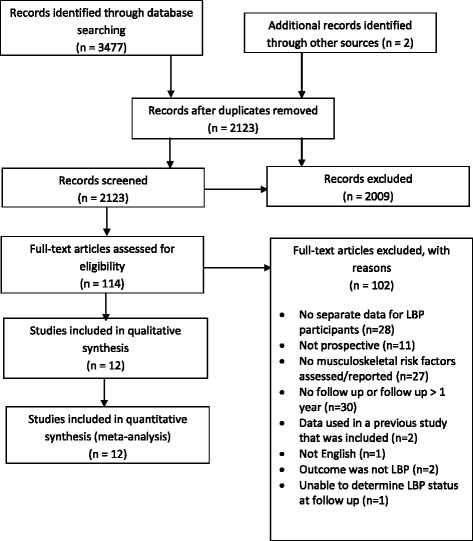
PRISMA flow diagram

Searchers retrieved a total of 3479 citations of which 114 were appropriate for full text review. After review, 12 articles were included while 102 were rejected based on exclusion criteria (Fig. 1). A list of full text articles with individual reasons for exclusion is included as a supplementary file (Additional file 2).

### Characteristics of included studies

The 12 articles [29–40] investigating musculoskeletal risk factors in the development of LBP included a total of 5459 participants (Table 1). All included studies had a follow-up period of 12 months with the exception of Milgrom et al. [37] where the follow-up period was 14 weeks. It was not possible to precisely determine the mean duration between the physical examination of participants and the development of their symptoms throughout the follow-up period, due to absence of this information. Two studies did not state whether participants had a history of LBP [30, 34], while six included participants with a history of LBP [29, 31, 32, 35, 38, 40], with two of these studies stating that those participants with a history of LBP were pain free at baseline [32, 38]. The four remaining studies only included participants with no recent history of LBP [33, 36, 37, 39].

**Table 1:** Summary of included studies (*n* = 12)

Study	Participants	Description of LBP/determination of a previous episode	Outcome measure (e.g. survey)	# that developed LBP in follow up year
Adams et al [29]	*n* = 403 Mean age (yr) = 27 Population = Health care workers Gender = 92% F Region: United Kingdom	History of LBP: *n* = 141 Pain free at baseline: not stated	Self-administered postal questionnaire, *n* = 399 (1% percent lost in follow up period)	*n* = 159 (39.5%) first time LBP
Biering-Sorensen et al [30]	*n* = 928 Mean age (yr) = not stated, range 30-60 Population = community members Gender = 52% F Region: Denmark	History of LBP: not stated Pain free at baseline: not stated	Self-administered postal questionnaire or telephone, *n* = 920 (1% percent lost in follow up period)	*n* = 170 (M), 185 (F) [recurrence or persistence of LBP]; *n* = 28 (M), 30 (F) [first time LBP 6.3%]
Feldman et al [31]	*n* = 810 Mean age (yr) = 14.1 Population = Adolescent students Gender = 47% F Region: Canada	History of LBP: *n* = 125 Pain free at baseline: not stated	Self-administered questionnaire, n = 502 (38% lost in follow up period)	*n* = 65 developed first time LBP (17.2%))
Fortin et al [40]	*n* = 99 Mean age (yr) = 47.3 Population = Monozygotic twins Gender = 0% F Region: Finland	History of LBP: 68 Pain free at baseline: not stated	Numeric pain scale, *n* = 98 (1% lost in follow up period)	*n* = 68 (recurrence or persistence of LBP, 69.4%)
Gibbons et al [32]	*n* = 130 Mean age (years) = 48 Population = Twins Gender = 0% F Region: Finland	History of LBP: *n* = 85 (in past year) Pain free at baseline: yes	Telephone, *n* = 128 (2% lost in follow up period)	*n* = 13 developed first time LBP (out of 43 that reported no history of LBP at baseline, 30.2%)); *n* = not reported for those 85 participants that declared a history of LBP in 12/12 before baseline testing
Kanchanomai et al [33]	*n* = 684 Mean age (years) = 19.4 Population = University students Gender = 74% F Region: Thailand	History of LBP: n = 0 (in past 3 months) Pain free at baseline: yes	Self-administered questionnaire, *n* = 524 (23% lost in follow up period)	*n* = 160 (23.4%) developed first time LBP
Kountouris et al [34]	*n* = 23 Mean age (years) = 24 Population = Elite fast bowlers Gender = 0% F Region: Australia	History of LBP: *n* = not stated Pain free at baseline: yes	Sports Medicine Physician and MRI, *n* = 23 (0% lost in follow up period)	*n* = 18 developed first time LBP (72.3%)
Kujala et al [35]	*n* = 138 Mean age (years) = not stated, range 10.3-13.3 Population = athletes and non-athletes Gender = not stated, 56% F at follow up Region: Finland	History of LBP: *n* = 28 (in past year) Pain free at baseline: not stated	Self-administered postal questionnaire, *n* = 119 (14% lost in follow up period)	*n* = 24 (11 recurrence of LBP [39.3%], 13 first time LBP [11.8%)
Luoto et al [36]	*n* = 167 Mean age (years) = not stated Population = blue and white collar workers Gender = 56% F Region: Finland	History of LBP: *n* = 0 (in past year) Pain free at baseline: yes	Nordic questionnaire, *n* = 126 (25% lost in follow up period)	*n* = 33 developed first time LBP (19.8%)
Milgrom et al [37]	*n* = 395 Mean age (years) = 18 Population = infantry recruits Gender = 0% F Region: Israel	History of LBP: *n* = 0 Pain free at baseline: not stated	Physical examination, *n* = not clear how many were lost in follow up period	*n* = 40 developed first time LBP (10.1%)
Nissinen et al [39]	*n* = 859 Mean age (years) = 12.8 Population = School children Gender = 48% F Region: Finland	History of LBP: *n* = 0 (in past year) Pain free at baseline	Self-administered standardised pain questionnaire, *n* = 859 (0% lost in follow up period)	*n* = 151 developed first time LBP (17.6%)
Van Nieuwenhuyse et al [38]	*n* = 823 Mean age (years) = 26 Population = workers at health care and distribution facilities Gender = 60% F Region: Belgium	History of LBP: *n* = 337 (in past year) Pain free at baseline: *n* = 688 (4 of the participants with history of LBP reported LBP at baseline)	Self-administered questionnaire, *n* = 692 (16% lost in follow up period)	*n* = 34 developed first time LBP (out of 355 that reported no history of LBP at baseline, 9.6%), *n* = 52 (out of 337 that reported LBP in 12/12 before testing, 15.4%)

*F* female, *M* male, *LBP* low back pain

Only a small number of studies provided details of how the outcome of LBP was measured (Table 1). One study [36] used the Nordic questionnaire while another study [33] just used a picture from the questionnaire and asked participants if they had experienced LBP. Fortin et al. [40] used a numeric pain scale to assess LBP. Most studies [29–32, 34, 35, 37–39] simply stated that a questionnaire was completed by participants at baseline and follow up. These studies asked participants, in an interview, physical examination, or questionnaire, whether or not they experienced LBP during the follow up period. It remains unclear if a valid and reliable measurement tool was used to assess the LBP of participants in these studies. In addition, no study identified the type or duration of LBP that participants developed during follow up.

### Study quality

Studies generally performed well in terms of quality with most satisfying the majority of the criteria on the quality appraisal tool (Table 2). The criterion regarding bias could not be assessed in the majority of cases due to lack of reporting of steps involved in participant assessment and management. Additionally, the generalisability of results of included studies was difficult to determine as there was a lack of reporting of cultural and ethical characteristics of the study populations, however, geographical information is reported in Table 1.

**Table 2:** Quality appraisal of included studies

	Adams et al [29]	Biering-Sorensen et al [30]	Feldman et al [31]	Fortin et al [40]	Gibbons et al [32]	Kanchanomai et al [33]	Kountouris et al [34]	Kujala et al [35]	Luoto et al [36]	Milgrom et al [37]	Nissinen et al [39]	Van Nieuwenhuyse et al [38]
1. Is the study relevant to the needs of the Project?	yes	yes	yes	yes	yes	yes	yes	yes	yes	yes	yes	yes
2. Does the paper address a clearly focused issue?	yes	yes	yes	yes	yes	yes	yes	yes	yes	yes	yes	yes
3. Is the choice of study method appropriate?	yes	yes	yes	yes	yes	yes	yes	yes	yes	yes	yes	yes
4. Is the population studied appropriate?	yes	yes	yes	yes	yes	yes	yes	yes	yes	yes	yes	yes
5. Is confounding and bias considered?	can’t tell	can’t tell	yes	yes	can’t tell	yes	yes	yes	can’t tell	can’t tell	can’t tell	can’t tell
6. Was follow up for long enough?	yes	yes	yes	yes	yes	yes	yes	yes	yes	can’t tell	yes	yes
7. Are tables/graphs adequately labelled and understandable?	yes	yes	yes	yes	yes	yes	yes	yes	yes	yes	yes	yes
8. Are you confident with the authors' choice and use of statistical methods, if employed?	yes	yes	yes	yes	yes	yes	yes	yes	yes	yes	yes	yes
9. What are the results of this piece of research? Are the authors' conclusions adequately supported by the information cited?	yes	yes	yes	yes	yes	yes	yes	yes	yes	yes	yes	yes
10. Can the results be applied to the local situation?	can’t tell	can’t tell	can’t tell	can’t tell	can’t tell	can’t tell	can’t tell	can’t tell	can’t tell	can’t tell	Can’t tell	can’t tell
11. Were all important outcomes/results considered?	yes	yes	yes	yes	yes	no	yes	no	no	yes	yes	yes
12. Is any cost information provided?	no	yes	no	no	no	yes	yes	yes	yes	no	yes	yes
13. Accept for further use as Type IV evidence?	yes	yes	yes	yes	yes	yes	yes	yes	yes	yes	yes	yes

### Meta-analyses

Due to studies measuring different musculoskeletal risk factors a combined meta-analysis was not appropriate. Individual meta-analyses were conducted based on risk factors common between studies. Meta-analyses investigating the following 12 musculoskeletal risk factors were conducted: lumbar extension range of motion (ROM), quadriceps flexibility, fingertip to floor distance, lumbar flexion ROM, lumbar lordosis, back muscle strength, back muscle endurance, abdominal strength, lateral bending ROM, erector spinae cross-sectional area (CSA), quadratus lumborum CSA, and hamstring flexibility.

**Fig. 2:**
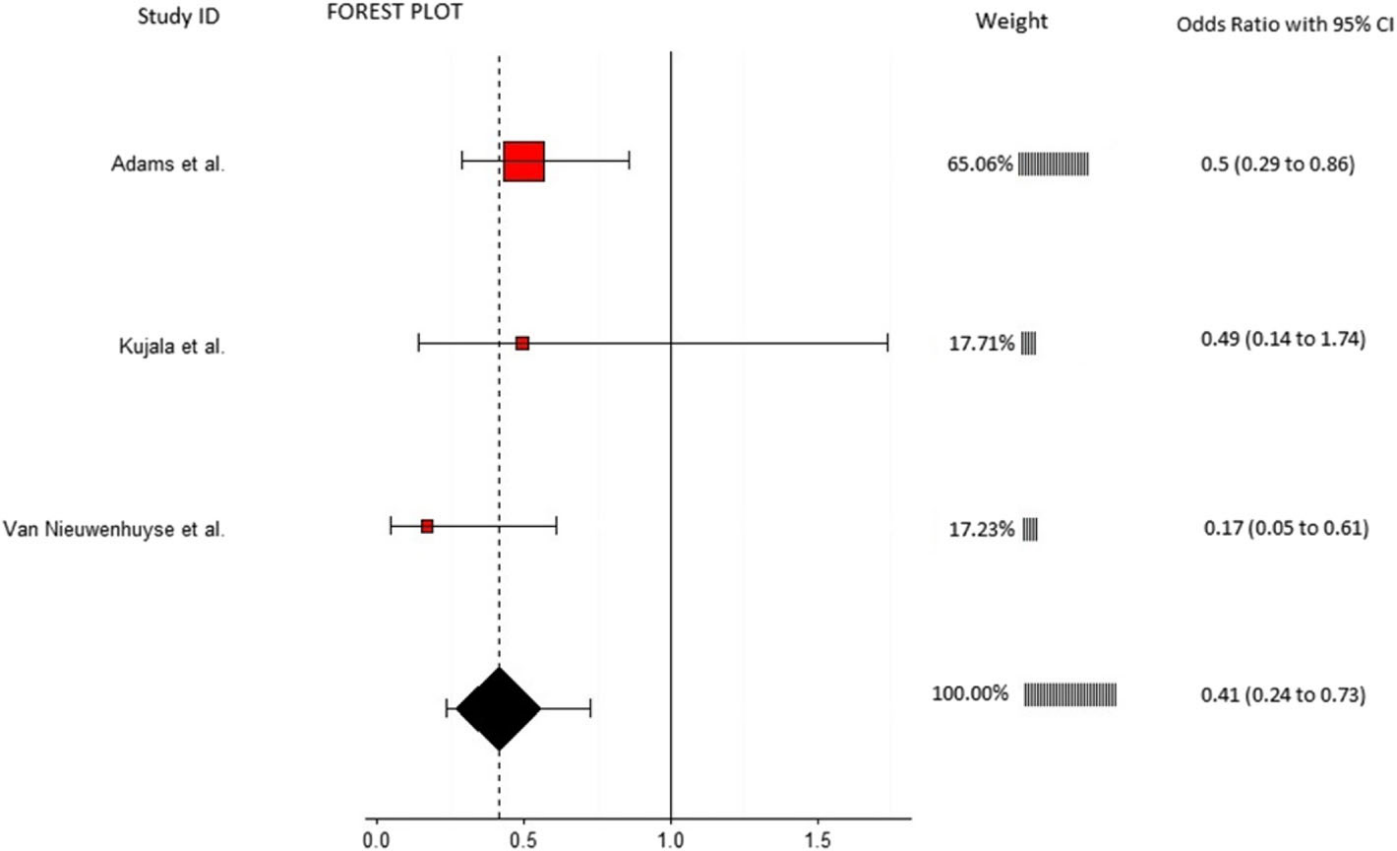
Annotated forest plot for lateral flexion range of motion and LBP

Three studies involving 1364 participant provided data for lateral flexion ROM and were eligible for inclusion in this meta-analysis (Fig. 2). Mean ages in included studies varied from 10 [35] to 27 years [29]. All three studies assessed lateral flexion ROM by measuring the distance that participants could slide their hand down their ipsilateral thigh, starting from an upright position to the point that pain occurred or further motion could not be achieved (Table 3). The analysis revealed a significant association between reduced lateral flexion ROM and the development of LBP (OR = 0.41, 95% CI 0.24-0.73, *p* = 0.002) with a low and non-significant amount of heterogeneity present (I^2^ = 15.9%, *p* = 0.304). Alternatively, this can be expressed as an OR of 2.44 (1/0.41) which means that those participants with limited lateral flexion ROM have a 144% greater likelihood of developing LBP.

**Table 3:** Overview of significant and nonsignificant risk factors in meta-analyses

Risk Factor	Study	Measurement technique	Effect size (95% CI)	Weight
Lateral flexion range of motion (ROM)	Adams et al [29]	Equipment: 3Space Isotrak device Method: Sensors on sacrum and L1 spinous process measured degree change between upright standing and max lateral flexion	0.50 (0.29-0.86)	65.06
	Kujala et al [35]	Equipment: Tape measure Methods: Difference between middle finger position on ipsilateral thigh to most distal position of middle finger achieved in max lateral flexion	0.49 (0.14-1.74)	17.71
	Van Nieuwenhuyse et al [38]	Equipment: Tape measure Methods: Difference between middle finger position on ipsilateral thigh to most distal position of middle finger achieved in max lateral flexion	0.17 (0.05-0.61)	17.23
	Overall effect size (I-squared = 15.9%, *p* = 0.304)		0.41 (0.24-0.73, *p* = 0.002)	100
Lumbar lordosis	Adams et al [29]	Equipment: 3Space Isotrak device Method: Sensors on sacrum and L1 spinous process measured degrees of lordosis in upright standing	0.56 (0.38-0.83)	35.48
	Milgrom et al [37]	Equipment: Cybex (EDI 320) inclinometer Method: Inclinometer place over spinous process of L4 and lordosis angle measured relative to horizontal	0.88 (0.48-1.62)	18.37
	Nissinen et al [39]	Equipment: Spinal pantograph Method: Spinal pantograph used to measure lordosis angle in an upright standing	0.84 (0.61-1.16)	46.15
	Overall effect size (I-squared = 29.7%, *p* = 0.241)		0.73 (0.55-0.98, *p* = 0.034)	100
Hamstring flexibility	Feldman et al [31]	Equipment: Goniometer Method: Supine position with hip at 90°, ipsilateral knee extended from 90° of flexion	0.96 (0.94-0.98)	99.92
	Kujala et al [35]	Equipment: Hydrogoniometer Method: Straight leg raise	0.70 (0.20-2.45)	0.04
	Van Nieuwenhuyse et al [38]	Equipment: Inclinometer Method: Straight leg raise	1.00 (0.31-3.2)	0.04
	Overall effect size (I-squared = 0%, *p* = 0.883)		0.96 (0.94-0.98, *p* = 0.001)	100
Back muscle strength	Biering-Sorensen et al [30]	Equipment: Strain gauge dynamometer Methods: Device attached to shoulders of participant and the MVC of 3 attempts of extension in upright standing	1.49 (0.70-3.16)	71.35
	Gibbons et al [32]	Equipment: not clear Methods: Max isokinetic strength from forward flexion to upright standing	1.81 (0.55-5.93)	28.65
	Overall effect size (I-squared = 0%, *p* = 0.788)		1.58 (0.83-2.97, *p* = 0.160)	100
Back muscle fatigability	Adams et al [29]	Equipment: Stopwatch Methods: Biering-Sorensen test	0.80 (0.60-1.07)	42.59
	Biering-Sorensen et al [30]	Equipment: Stopwatch Methods: Biering-Sorensen test	0.42 (0.16-1.14)	16.57
	Gibbons et al [32]	Equipment: Stopwatch Methods: Biering-Sorensen test	0.85 (0.26-2.77)	12.97
	Kujala et al [35]	Equipment: Stopwatch Methods: Biering-Sorensen test	1.87 (0.53-6.56)	11.80
	Luoto et al [36]	Equipment: Stopwatch Methods: Biering-Sorensen test	0.29 (0.11-0.81)	16.06
	Overall effect size (I-squared = 41.2%, *p* = 0.147)		0.68 (0.42-1.12 *p* = 0.160)	100
Lumbar flexion range of motion (ROM)	Adams et al [29]	Equipment: 3Space Isotrak device Method: Sensors on sacrum and L1 spinous process measured degree change between upright standing and max forward flexion sitting with legs extended	1.25 (0.94-1.67)	50.92
	Biering-Sorensen et al [30]	Equipment: Tape measure Method: Modified Schober	2.59 (1.23-5.46)	19.98
	Feldman et al [31]	Equipment: Tape measure Method: Schober	0.93 (0.45-1.93)	20.43
	Kujala et al [35]	Equipment: Tape measure Method: Modified Schober	0.88 (0.25-3.07)	8.67
	Overall effect size (I-squared = 34%, *p* = 0.209)		1.32 (0.89-1.96, *p* = 0.167)	100
Lumbar extension ROM	Adams et al [29]	Equipment: 3Space Isotrak device Method: Sensors on sacrum and L1 spinous process measured degree change between upright standing and max extension in prone position	0.95 (0.67-1.34)	45.69
	Kujala et al [35]	Equipment: Draughtsman’s flexible curve Method: Devices placed on spinous process of S2, L4, and T12 in prone position with max extension. Curve traced on paper then angle in degrees measured.	1.19 (0.34-4.14)	22.76
	Van Nieuwenhuyse et al [38]	Equipment: none Method: passive extension of lower back measured as presence or absence of pain	0.29 (0.12-0.70)	31.55
	Overall effect size (I-squared = 68.7%, *p* = 0.041)		0.69 (0.31-1.55, *p* = 0.367)	100
Isometric abdominal strength	Biering-Sorensen et al [30] 0	Equipment: Strain gauge dynamometer Methods: Device attached to shoulders of participant and the MVC of 3 attempts of flexion in upright standing	1.20 (0.56-2.53)	16.62
	Feldman et al [31]	Equipment: Hand held myometer Methods: Sit-up, stop midway then resistance applied to the sternum. Max force that participant could hold in that position recorded	0.96 (0.69-1.34)	83.38
	Overall effect size (I-squared = 0%, *p* = 0.602)		1.00 (0.73-1.35, *p* = 0.976)	100
Fingertip to floor distance	Biering-Sorensen et al [30]	Equipment: Tape measure Method: Distance from tips of the middle fingers to the ground during max forward bending with feet together and knees extended	0.96 (0.40-2.28)	0.13
	Feldman et al [31]	Equipment: Sit and reach box Method: Sitting with hips flexed and knees extended	1.00 (0.97-1.03)	99.53
	Kujala et al [35]	Equipment: Tape measure Method: Distance from tips of the middle fingers to the ground during max forward bending with feet together and knees extended	2.65 (0.75-9.37)	0.06
	Van Nieuwenhuyse et al [38]	Equipment: Tape measure Method: Distance from tips of the middle fingers to the ground during max forward bending with feet together and knees extended	1.00 (0.55-1.81)	0.28
	Overall effect size (I-squared = 0%, *p* = 0.515)		1.00 (0.97-1.03, *p* = 0.973)	100
Quadriceps flexibility	Feldman et al [31]	Equipment: Goniometer Method: Degrees of knee flexion in a prone position	1.02 (0.92-1.13)	62.13
	Kanchanomai et al [33]	Equipment: Goniometer Method: Stationary arm of device aligned with the lateral midline of the thigh, fulcrum is placed over the lateral epicondyle of the femur and the moving arm is aligned with the lateral midline of the fibula. Smaller degrees of knee flexion equated to tighter quadriceps	1.71 (1.03-2.84)	37.87
	Overall effect size (I-squared = 73.9%, *p* = 0.050)		1.24 (0.76-2.03, *p* = 0.389)	100
Erector spinae CSA	Fortin et al [40]	Equipment: 1.5 Tesla Magnetom SP 4000 magnetic resonance imager Method: T2-weighted techniques at L3-4 and L5-S1	-0.09 (-0.48-0.31)	27.25
	Gibbons et al [32]	Equipment: 1.5 tesla Magnetom magnetic resonance imager Method: Slice thickness was 3 mm and gaps between the slices 0.3 mm at the L3-4 level.	0.00 (-0.65-0.65)	72.75
	Overall effect size (I-squared = 0%, *p* = 0.823)		-0.06 (-0.40-0.28, *p* = 0.722)	100
Quadratus lumborum CSA	Gibbons et al [32]	Equipment: 1.5 tesla Magnetom magnetic resonance imager Method: Slice thickness was 3 mm and gaps between the slices 0.3 mm at the L3-4 level.	2.96 (0.89-9.86)	82.85
	Kountouris et al [34]	Equipment: MRI and imaging software Method: Axial MR images measured at L2 and L4 levels	0.55 (0.03-10.37)	17.15
	Overall effect size (I-squared = 8%, *p* = 0.297)		2.22 (0.63-7.74, *p* = 0.212)	100

*MVC* max voluntary contraction, *CSA* cross sectional area, *MRI* magnetic resonance imaging

**Fig. 3:**
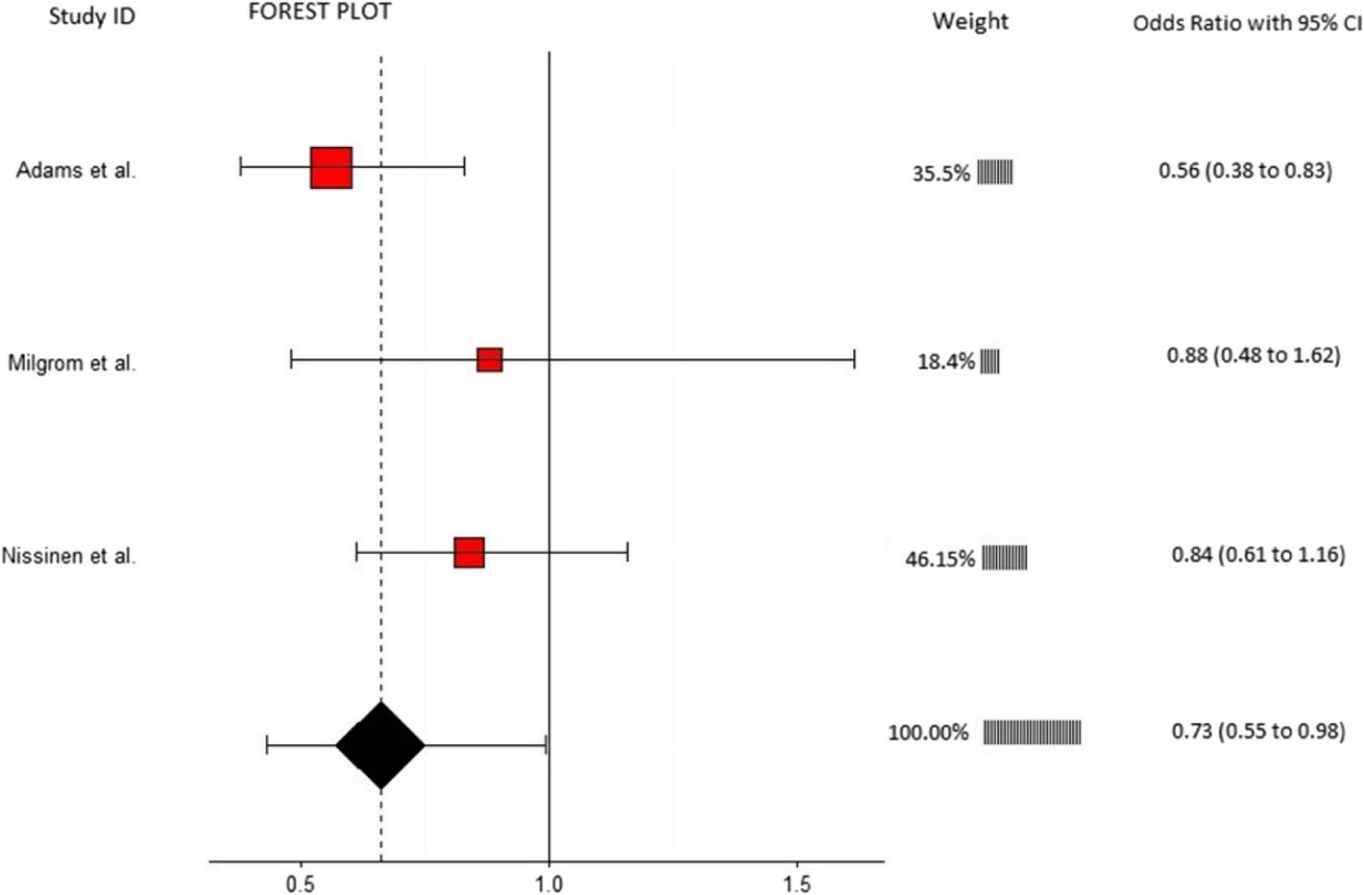
Annotated forest plot for lumbar lordosis and LBP

Three studies involving 1657 participants provided data for lumbar lordosis and were eligible for inclusion in this meta-analysis (Fig. 3). Mean ages in included studies varied from 12.8 [39] to 27 years [29]. Each study used a different device to measure lumbar lordosis with Adams et al. [29] and Milgrom et al. [37] using different reference points, from which the angle was measured, of L1 and L4 respectively (Table 3). Nissinen et al. [39] did not report a reference point. The analysis revealed a significant association between a reduction in lumbar lordosis and the risk of developing LBP (OR = 0.73, 95% CI 0.55-0.98, *p* = 0.034). Alternatively, this can be expressed as an OR of 1.37 (1/0.73) or a 37% greater likelihood of developing LBP in people with restricted lumbar lordosis. No significant heterogeneity was detected (I^2^ = 29.7%, *p* = 0.241).

**Fig. 4:**
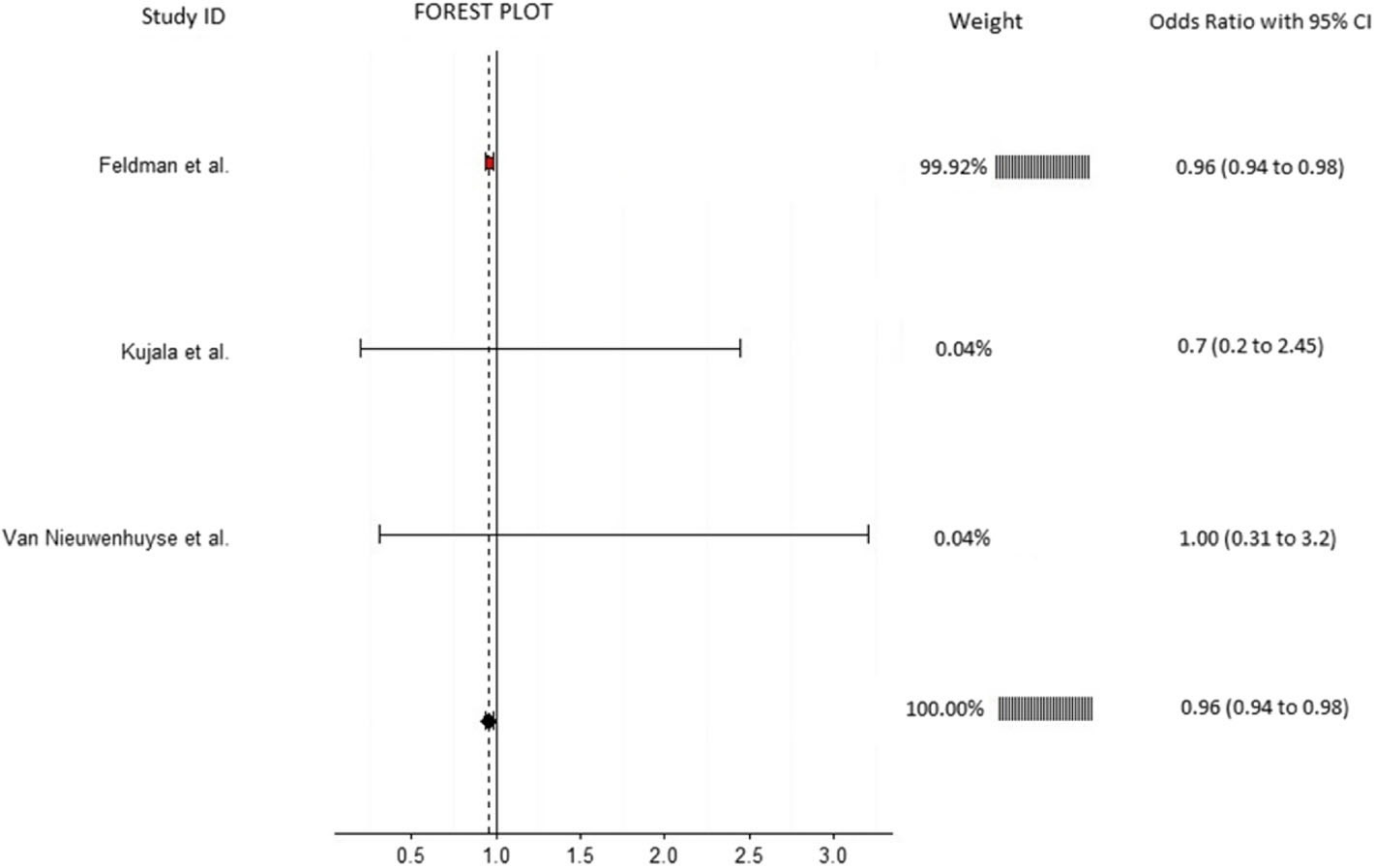
Annotated forest plot for hamstring flexibility and LBP

Three studies involving 1771 participants provided data for hamstring ROM and were eligible for inclusion in this meta-analysis (Fig. 4). Ages in included studies ranged from 10 [35] to 26 years [38]. Kujala et al. [35] and Van Nieuwenhuyse et al. [38] used the straight leg raise test, whereas Feldman et al. [31] used the passive knee extension test (Table 3). The analysis revealed a significant association between restricted hamstring ROM and the risk of developing LBP (OR = 0.96, 95% CI 0.94-0.98, *p* = 0.001) which is equivalent to an OR of 1.04 (1/0.96) or a 4% greater likelihood of developing LBP in those participants with limited hamstring ROM. No significant heterogeneity was detected (I^2^ = 0%, *p* = 0.883).

Meta-analyses on all other musculoskeletal risk factors showed non-significant results and are included as part of Table 3. Fully annotated forest plots for the non-significant meta-analyses (Additional file 3) are included as a supplementary file. The effect sizes for all meta-analyses were calculated as odds ratios except for the erector spinae meta-analysis, which was calculated as a standard mean difference because the figures were reported as continuous data.

Additional musculoskeletal risk factors were investigated by the studies included in this review; however, were not combined in meta-analyses because they were only measured in one study (Additional file 4). Some studies did measure the same risk factors as other studies, but did not report the results within their study. In such instances and when other additional information was required, the authors were contacted and the necessary data requested, but no additional data were provided except for information from Adams et al. [29] which aided in the calculation of the odds ratios for this study.

## Discussion

Our systematic review of the literature found 12 prospective studies eligible for inclusion, most of which demonstrated moderate methodological quality on the appraisal tool (Table 2). These studies investigated a range of musculoskeletal risk factors, most of which related to the lower back and pelvic region, for the development of LBP with multiple studies allowing meta-analyses on the following risk factors: lumbar extension ROM, quadriceps flexibility, fingertip to floor distance, lumbar flexion ROM, lumbar lordosis, back muscle strength, back muscle endurance, abdominal strength, lateral bending ROM, erector spinae CSA, quadratus lumborum CSA, and hamstring flexibility. Based on the results of our meta-analyses, restrictions in lateral flexion ROM, hamstring flexibility, and lumbar lordosis were found to increase the risk of participants developing LBP. To the authors knowledge these are the first meta-analyses of these musculoskeletal risk factors to demonstrate significant prospective relationships. However, due to the mixed populations of the included studies and the close proximity of the upper band of the confidence interval for the lumbar lordosis and hamstring ROM meta-analyses, some caution is advised when interpreting these significant meta-analyses. In addition, due to the small number of studies included the meta-analyses of back muscle strength, isometric abdominal strength, quadriceps flexibility, erector spinae CSA, and quadratus lumborum CSA the results from these analyses can only be considered as a summary measure and definitive conclusions cannot be drawn. The low number of included studies have resulted in these meta-analyses lacking power and generalisability [27]. Furthermore when interpreting all of the results of this systematic review, the inclusion of study populations with and without a history of LBP must be considered. Although some studies included only participants without a history of recent LBP [33, 36, 37, 39], a number included some participants who have previously experienced the condition [29, 31, 32, 35, 38, 40]. It is important that the results of these studies are interpreted in context of this, as previous LBP has been shown to be a risk factor for the development of LBP [23], making the establishment of a cause and effect relationship difficult. Nevertheless, as the majority of studies demonstrated a consistent direction of association and there are extremely limited data available, the results of the meta-analyses are important to improve the collective understanding of the nature of LBP.

Lateral flexion, in addition to sagittal plane motion, facilitates the spine to absorb force [41] and it is perhaps due to this restriction in motion and therefore stiffness in the lower back that participants developed LBP [30, 42, 43]. We found a reduction in lateral flexion ROM to be prospectively associated with the development of LBP, which concurs with the results reported previously in a systematic review of case control studies [44].

As with lateral flexion of the lower back, lumbar lordosis is responsible for absorbing force [29]. A previous systematic review of retrospective articles found no difference between the degree of lumbar lordosis in those with and without LBP [44]. Our findings are contrary to this and suggest that a reduced lumbar lordosis is an important musculoskeletal risk factor for the development of LBP. The conflicting findings may be explained by the differences between the study types or participants included in the reviews, or that the degree of lumbar lordosis may change in response to LBP developing so that symptomatic relief may be achieved through adopting a position that is similar to those who are asymptomatic. Furthermore our results may also have been affected by how lumbar lordosis was measured in the included studies, particularly as the reference point for measuring lumbar lordosis differed between some of the included studies (Table 3), which may have influenced the degree of lordosis measured in some participants within these studies [29, 37, 39]. It is also important to note that other sagittal plane parameters, such as sacral slope and pelvic incidence, can influence the degree of lordosis and these parameters themselves, although not reported in the included studies, could be associated with the development of LBP.

Restricted hamstring ROM has been linked to reduced lumbar lordosis [45] and it is perhaps through this mechanism and subsequent stiffness in the lower back that a significant association was demonstrated. Although the results of our meta-analysis support this, these need to be interpreted in light of the dominance of one study in the analysis [31] and the fact that the confidence intervals are close to the point of no effect.

The meta-analyses on a number of musculoskeletal risk factors including back muscle endurance, lumbar extension ROM, quadriceps flexibility, and lumbar flexion ROM were not significant. This may be due to the moderate to high heterogeneity between the studies and may be partly explained by the inclusion of participants with and without a history of LBP as previously discussed [29, 31, 35]. Additionally, there were notable differences in demographics of study populations, measurement techniques, and settings in which the studies were conducted further highlighting the diverse nature of the literature on LBP.

Contrastingly, meta-analyses on finger-tip to floor distance, back muscle strength, abdominal strength, CSA of erector spinae and quadratus lumborum were also non-significant but had no or minimal heterogeneity. Supporting this is that no study, in these meta-analyses, reported that these risk factors were significant predictors for the development of LBP. This indicates that these factors are less likely to be predictors for the development of LBP. The mixed populations and measurement techniques, as well as the small number of included studies, indicate that these results should be interpreted with caution and that additional research is needed before these risk factors can be confidently excluded as possible predictors of LBP.

Clinically, restriction in lateral bending ROM of the lumbar region, limited lumbar lordosis, and tight hamstring muscles can be used as predictive musculoskeletal risk factors for the development of LBP. Advising patients of the relationship of these musculoskeletal measures to LBP plus the therapy options to modify these risk factors may potentially serve to prevent the development of LBP. However, the risk factors that were found to be nonsignificant for the development of LBP require more investigation before they can be confidently recommended as risk factors that should be measured in clinical practice.

Previous research [46–48], has identified that the cause of LBP is likely to be multifactorial with physical, psychological, environmental, and demographic factors potentially contributing to the development of LBP in differing proportions between individuals. This makes the identification of risk factors which are predictive of LBP from one domain challenging in heterogeneous populations. Supporting this are the findings of a previous study investigating interventions for LBP which demonstrated that patients’ response to interventions for the treatment of LBP may be more consistent when applied to a homogenous population. For example, while foot orthoses have been shown to have little effect on LBP in the general population [49], Castro-Mendez and colleagues [50] found that participants with LBP and excessive foot pronation were more likely to improve with the use of custom foot orthoses compared to a control intervention. These findings suggest that musculoskeletal risk factors may have a greater contribution to the development of LBP in certain subgroups.

The secondary aims of this systematic review included the identification of the type and duration of LBP that participants developed in the included studies. None of the included studies stated whether participants developed specific or non-specific LBP, nor was the duration of LBP identified. Identification of the type and duration of LBP may provide further guidance in the management of LBP and could lead to a clearer definition of LBP. Additionally, and due to the inconsistent definitions of what constitutes LBP, it may also clarify if certain risk factors truly do predict the development of LBP. The authors also suggest that regular reporting of LBP symptoms is needed to prevent recall bias and accurately categorise the type and duration of LBP.

### Limitations

This systematic review is not without limitations. Although our electronic search encompassed a range of databases and our manual search a number of additional sources we only included articles published in English. The authors decided to limit the maximum follow-up period to 12 months because of the potential for extraneous variables to influence the development of LBP over an extended period of time. However, because it is not known if this would happen it is worth considering that additional risk factors may be have been discovered or the effect sizes of included predictor variables may have differed if studies of longer durations were included. Likewise, the short follow up time of 14 weeks in the Milgrom et al. [37] study may have not been long enough for participants to develop LBP therefore influencing the effect size of risk factors measured. Consideration of the limitations of this systematic review and those of the individual studies is recommended when interpreting the results.

## Conclusion

In summary, we found that a restriction in lateral flexion and hamstring ROM, as well as, reduced lumbar lordosis were associated with an increased risk of developing LBP over a 12 month period. The majority of musculoskeletal risk factors investigated in the literature relate to the lower back region. Consequently, future research should aim to measure additional lower limb musculoskeletal risk factors, adopt a standardised definition of LBP, provide raw data so results can be meaningfully pooled between studies, and only include participants with no history of LBP.

## Additional files

Additional file 1: Detailed search strategy. (DOCX 16 kb)

Additional file 2: List of full text articles excluded with reasons. (DOCX 27 kb)

Additional file 3: Fully annotated forest plots for non-significant meta-analyses. (DOCX 449 kb)

Additional file 4: Overview of risk factors measured in single studies. (DOCX 20 kb)
