# Supplementary material for: Restriction in lateral bending range of motion, lumbar lordosis, and hamstring flexibility predicts the development of low back pain: a systematic review of prospective cohort studies
Source: BMC Musculoskelet Disord. 2017 May 5;18:179. doi: 10.1186/s12891-017-1534-0 (PMC5418732; doi:10.1186/s12891-017-1534-0)
Supplement: Supplementary file 1 — Detailed search strategy. (DOCX 16 kb) [file 12891_2017_1534_MOESM1_ESM.docx]

| MEDLINE, EMBASE, and AMED search strategy (from inception to Feb 2016) |
| --- |
| 1. exp Low Back Pain/ |
| 2. chronic low* back pain.mp. |
| 3. nonspecific low* back pain.mp. |
| 4. low* back trouble.mp. |
| 5. LBT.mp. |
| 6. LBP.mp. |
| 7. back.mp. |
| 8. 1 or 2 or 3 or 4 or 5 or 6 or 7 |
| 9. biomechanic*.mp. |
| 10. musc?l*.mp. |
| 11. strength.mp. |
| 12. endurance.mp. |
| 13. physical examination*. |
| 14. core stability.mp. |
| 15. physical measurement*.mp. |
| 16. hamstring*.mp. |
| 17. flexib*.mp. |
| 18. range of motion.mp. |
| 19. lower extremity.mp. |
| 20. f??t.mp. |
| 21. ankle.mp. |
| 22. knee.mp. |
| 23. hip.mp. |
| 24. 9 or 10 or 11 or 12 or 13 or 14 or 15 or 16 or 17 or 18 or 19 or 20 or 21 or 22 or 23 |
| 25. risk factor*.mp. |
| 26. risk indicator*.mp. |
| 27. predict*.mp. |
| 28. prognostic factor*.mp. |
| 29. 25 or 26 or 27 or 28 |
| 30. cohort.mp. |
| 31. longitudinal.mp |
| 32. prospective.mp. |
| 33. follow-up.mp. |
| 34. 30 or 31 or 32 or 33 |
| 35. 8 and 24 and 29 and 34 |

**Additional file 1. Search strategy**

| CINAHL, SPORTDiscus, and Cochrane (from inception to Feb 2016) |
| --- |
| (Low* back pain or chronic low* back pain or nonspecific low* back pain or low* back trouble or LBT or LBP or back) AND (Biomechanic* or musc?l* or strength or endurance or physical examination* or physical measurement* or core stability or flexib* or hamstring* or range of motion or lower extremity or f??t or ankle or knee or hip) AND (prospective or cohort or longitudinal or follow-up) AND (risk factor* or risk indicator* or predict* or prognostic factor*) |
