# Supplementary material for: Restriction in lateral bending range of motion, lumbar lordosis, and hamstring flexibility predicts the development of low back pain: a systematic review of prospective cohort studies
Source: BMC Musculoskelet Disord. 2017 May 5;18:179. doi: 10.1186/s12891-017-1534-0 (PMC5418732; doi:10.1186/s12891-017-1534-0)
Supplement: Supplementary file 2 — List of full text articles excluded with reasons. (DOCX 27 kb) [file 12891_2017_1534_MOESM2_ESM.docx]

| **Additional file 2. Excluded full text articles with reasons** | | | |
| --- | --- | --- | --- |
|  | **Author** | **Title** | **Grounds for Exclusion** |
|  | Andersen et al 2012 | Spreading of chronic pain between body regions: Prospective cohort study among health care workers. European Journal of Pain 2012;16(10):1437-43 | No musculoskeletal risk factors assessed/reported |
|  | Balague et al 2010 | The association between isoinertial trunk muscle performance and low back pain in male adolescents. European Spine Journal 2010;19(4):624-32 | No separate data for LBP participants |
|  | Barnekow-Bergkvis et al 1998 | Determinants of self-reported neck-shoulder and low back symptoms in a general population. Spine;23(2):235:43 | No musculoskeletal risk factors assessed/reported |
|  | Battie et al 1989 | Isometric lifting strength as a predictor of industrial back pain reports. Spine;14(8):851-6 | No separate data for LBP participants |
|  | Battie et al 1990 | Anthropometric and clinical measures as predictors of back pain complaints in industry: a prospective study. Journal of Spinal Disorders;3(3):195-204 | No follow up or follow up > 1 year |
|  | Battie et al 1990 | The role of spinal flexibility in back pain complaints within industry. A prospective study. Spine;15(8):768-73 | No follow up or follow up > 1 year |
|  | Bergstrom et al 2007 | Risk factors for new episodes of sick leave due to neck or back pain in a working population. A prospective study with an 18-month and a three-year follow-up. Occupational and Environmental Medicine;64(4):279-87 | No musculoskeletal risk factors assessed/reported |
|  | Biering-Sorensen et al 1984 | A one-year prospective study of low back trouble in a general population. The prognostic value of low back history and physical measurements. Danish Medical Journal;31(5):362-75 | Data used in a previous study that was included |
|  | Biering-Sorensen et al 1986 | Medical, social and occupational history as risk indicators for low-back trouble in a general population. Spine;11(7):720-5 | No musculoskeletal risk factors assessed/reported |
|  | Biering-Sorensen et al 1989 | Risk indicators for low back trouble. Scandinavian Journal of Rehabilitation Medicine;21(3):151-7 | Data used in a previous study that was included |
|  | Bigos et al 1992 | A prospective evaluation of preemployment screening methods for acute industrial back pain. Spine;17(8):922-6 | No separate data for LBP participants |
|  | Bigos et al 1992 | A longitudinal, prospective study of industrial back injury reporting. Clinical Orthopaedics and related research;279:21-34 | No separate data for LBP participants |
|  | Bridger et al 2013 | Sustaining person-environment fit with a changing workforce. Ergonomics;56(3):565-77 | No musculoskeletal risk factors assessed/reported |
|  | Broniecki et al 2011 | Pre-employment risk factors for back, neck and shoulder musculoskeletal injuries and claims in ambulance officers. Journal of Musculoskeletal Research;14(1):1-10 | Not prospective |
|  | Brumitt et al 2013 | Lower extremity functional tests and risk of injury in division 3 collegiate athletes. International Journal of Sports Physical Therapy;8(3):216-27 | No musculoskeletal risk factors assessed/reported |
|  | Buist et al 2010 | Predictors of running-related injuries in novice runners enrolled in a systematic training program: a prospective cohort study. The American Journal of Sports Medicine;38(2):273-80 | No separate data for LBP |
|  | Cheung 2010 | The incidence of low back problems among nursing students in Hong Kong. Journal of Clinical Nursing;19(15):2355-62 | No musculoskeletal risk factors assessed/reported |
|  | Cholewicki et al 2005 | Delayed trunk muscle reflex responses increase the risk of low back injuries. Spine;30(23):2614-20 | No follow up or follow up > 1 year |
|  | Claeys et al 2015 | Young individuals with a more ankle-steered proprioceptive control strategy may develop mild non-specific low back pain. Journal of Electromyography and Kinesiology;25(2):329-38 | No follow up or follow up > 1 year |
|  | Coenen et al 2015 | The effect of the presence and characteristics of an outlying group on exposure–outcome associations. Scandinavian Journal of Work, Environment & Health;41(1):65-74 | No follow up or follow up > 1 year |
|  | Crill et al 2005 | Back strength and flexibility of EMS providers in practicing prehospital providers. Journal of Occupational Rehabilitation;15(2):105-11 | Not prospective |
|  | Cupisti et al 2007 | Injury survey in competitive sub-elite rhythmic gymnasts: results from a prospective controlled study. Journal of Sports Medicine & Physical Fitness;47(2):203-7 | No musculoskeletal risk factors assessed/reported |
|  | De Cuyper et al 1988 | The lordosis angle as a predictor of lumbar symptoms. A preliminary study. Acta Belgica – Medica Physica;11(1):21-30 | Not English |
|  | Dennis et al 2008 | Use of field-based tests to identify risk factors for injury to fast bowlers in cricket. British Journal of Sports Medicine;42(6):477-82 | No separate data for LBP |
|  | Dieck et al 1985 | An epidemiologic study of the relationship between postural asymmetry in the teen years and subsequent back and neck pain. Spine;10(10):872-7 | No musculoskeletal risk factors assessed/reported |
|  | Edmond et al 2005 | Vertebral deformity, back symptoms, and functional limitations among older women: The Framingham Study. Osteoporosis International;16(9):1086-95 | No musculoskeletal risk factors assessed/reported |
|  | El-Metwally et al 2007 | Risk factors for development of non-specific musculoskeletal pain in preteens and early adolescents: a prospective 1-year follow-up study. BMC Musculoskeletal Disorders;8:46 | No separate data for LBP |
|  | Ettinger et al 1992 | Contribution of vertebral deformities to chronic back pain and disability. The Study of Osteoporotic Fractures Research Group. Journal of Bone & Mineral Research;7(4):449-56 | Not prospective |
|  | Evans et al 2005 | Predictors of low back pain in young elite golfers: A preliminary study. Physical Therapy in Sport;6(3):122-30 | No separate data for LBP participants |
|  | Faber et al 2012 | Does muscle strength predict future musculoskeletal disorders and sickness absence? Occupational Medicine;62(1):41-6 | No separate data for LBP participants |
|  | Ferguson 2012 | Biomechanical, psychosocial and individual risk factors predicting low back functional impairment among furniture distribution employees. Clinical Biomechanics;27(2):117-23 | Outcome was not LBP |
|  | Hamberg-Van Reenen et al 2006 | Is an imbalance between physical capacity and exposure to work-related physical factors associated with low-back, neck or shoulder pain? Scandinavian Journal of Work, Environment & Health;32(3):190-7 | No musculoskeletal risk factors assessed/reported |
|  | Hamberg-Van Reenen et al 2006 | Physical capacity in relation to low back, neck, or shoulder pain in a working population. Occupational & Environmental Medicine;63(6):371-7 | No follow up or follow up > 1 year |
|  | Harbin et al 2005 | Post-offer, pre-placement testing in industry. American Journal of Industrial Medicine;47(4):296-307 | No musculoskeletal risk factors assessed/reported |
|  | Harreby et al 1999 | Risk factors for low back pain in a cohort of 1389 Danish school children: an epidemiologic study. European Spine Journal;8(6):444-50 | Not prospective |
|  | Hartvigsen et al 2006 | Physical and mental function and incident low back pain in seniors: a population-based two-year prospective study of 1387 Danish Twins aged 70 to 100 years. Spine;31(14):1628-32 | No musculoskeletal risk factors assessed/reported |
|  | Hebert et al 2014 | The relationship of lumbar multifidus muscle morphology to previous, current, and future low back pain: A 9-year population-based prospective cohort study. Spine;39(17):1417-25 | No musculoskeletal risk factors assessed/reported |
|  | Herin et al 2014 | Predictive risk factors for chronic regional and multisite musculoskeletal pain: A 5-year prospective study in a working population. Pain;155(5):937-43 | No musculoskeletal risk factors assessed/reported |
|  | Heydari et al 2010 | EMG analysis of lumbar paraspinal muscles as a predictor of the risk of low-back pain. European Spine Journal;19(7):1145-52 | No follow up or follow up > 1 year |
|  | Hick et al 2005 | Cross-sectional associations between trunk muscle composition, back pain, and physical function in the health, aging and body composition study. The Journals of Gerontology, Series A, Biological Sciences and Medical Science;60(7):882-7 | Not prospective |
|  | Hjelm et al 2012 | Injury risk factors in junior tennis players: a prospective 2-year study. Scandinavian Journal of Medicine and Science in Sport;22(1):40-8 | No musculoskeletal risk factors assessed/reported |
|  | Hoogendoorn et al 2000 | Flexion and rotation of the trunk and lifting at work are risk factors for low back pain: results of a prospective cohort study. Spine;25(23):3087-92 | No musculoskeletal risk factors assessed/reported |
|  | Hopper et al 1995 | Do selected kinanthropometric and performance variables predict injuries in female netball players? Journal of Sports Sciences;13(3):213-22 | No separate data for LBP |
|  | Husu et al 2012 | Predictive validity of health-related fitness tests on back pain and related disability: a 6-year follow-up study among high-functioning older adults. Journal of Physical Activity & Health;9(2):249-58 | No follow up or follow up > 1 year |
|  | Jackson et al 1998 | Relations of sit-up and sit-and-reach tests to low back pain in adults. Journal of Orthopaedic and Sports Physical Therapy;27(1):22-6 | No follow up or follow up > 1 year |
|  | Janwantanakul et al 2015 | A screening tool for non-specific low back  pain with disability in office workers: a 1-  year prospective cohort study. BMC Musculoskeletal Disorders;16:298 | No separate data for LBP |
|  | Kaaria et al 2010 | Pain and clinical findings in the low back: A study of industrial employees with 5-, 10-, and 28-year follow-ups. European Journal of Pain;14(7):759-63 | No follow up or follow up > 1 year |
|  | Klaber Moffett et al 1993 | A longitudinal study of low back pain  in student nurses. International Journal of Nursing Studies;30(3):197-212 | No separate data for LBP |
|  | Krause et al 2004 | Physical workload, ergonomic problems, and incidence of low back injury: a 7.5-year prospective study of San Francisco transit operators. American Journal of Industrial Medicine;46(6):570-85 | No musculoskeletal risk factors assessed/reported |
|  | Kujala et al 1997 | Lumbar mobility and low back pain during adolescence. A longitudinal three-year follow-up study in athletes and controls. American Journal of Sports Medicine;25(3):363-8 | No follow up or follow up > 1 year |
|  | Kujala et al 1996 | Physical loading and performance as predictors of back pain in healthy adults. A 5-year prospective study. European Journal of Applied Physiology & Occupational Physiology;73(5):452-8 | No follow up or follow up > 1 year |
|  | Larsen et al 2006 | Coping and back problems: a prospective observational study of Danish military recruits. Journal of Manipulative & Physiological Therapeutics;29(8):619-25 | No separate data for LBP participants |
|  | Lee et al 1999 | Trunk muscle weakness as a risk factor for low back pain. A 5-year prospective study. Spine;24(1):54-7 | No follow up or follow up > 1 year |
|  | Lee et al 1983 | Back on their feet. Predicting problems. Nursing Times;79(42):51-4 | No musculoskeletal risk factors assessed/reported |
|  | Leetun et al 2004 | Core stability measures as risk factors for lower extremity injury in athletes. Medicine & Science in Sports & Exercise;36(6):926-34 | No separate data for LBP |
|  | Legge et al 2013 | A new pre-employment functional capacity evaluation predicts longer-term risk of musculoskeletal injury in healthy workers: a prospective cohort study. Spine;38(25):2208-15 | No separate data for LBP |
|  | Lu et al 2014 | Efficacy of the revised NIOSH lifting equation to predict risk of low-back pain associated with manual lifting: a one-year prospective study. Human Factors;56(1):73-85 | No musculoskeletal risk factors assessed/reported |
|  | Luke et al 2002 | Determinants of injuries in young dancers. Medical Problems of Performing Artists;17(3):105-12 | No separate data for LBP |
|  | Makris et al 2014 | Risk factors for restricting back pain in older persons. Journal of the American Medical Directors Association;15(1):62-7 | No follow up or follow up > 1 year |
|  | Marras et al 2010 | Quantitative dynamic measures of physical exposure predict low back functional impairment. Spine;35(8):914-23 | Outcome was not LBP |
|  | Masset et al 1998 | Relation between functional characteristics of the trunk and the occurrence of low back pain. Associated risk factors. Spine;23(3):359-65 | No follow up or follow up > 1 year |
|  | McManus et al 2004 | Incidence and risk factors for injury in non-elite Australian Football. Journal of Science & Medicine in Sport;7(3):384-91 | No separate data for LBP |
|  | Menz et al 2013 | Foot posture, foot function and low back pain: the Framingham Foot Study. Rheumatology;52(12):2275-82 | Not prospective |
|  | Mikkelsson et al 2006 | Adolescent flexibility, endurance strength, and physical activity as predictors of adult tension neck, low back pain, and knee injury: a 25 year follow up study. British Journal of Sports Medicine;40(2):107-13 | No follow up or follow up > 1 year |
|  | Mitchell et al 2010 | Identification of modifiable personal factors that predict new-onset low back pain: a prospective study of female nursing students. Clinical Journal of Pain;26(4):275-83 | No separate data for LBP participants |
|  | Moseley 2014 | Impaired trunk muscle function in sub-acute neck pain: etiologic in the subsequent development of low back pain? Manual therapy;9(3):157-63 | No follow up or follow up > 1 year |
|  | Mostardi et al 1992 | Isokinetic lifting strength and occupational injury. A prospective study. Spine;17(2):189-93 | No follow up or follow up > 1 year |
|  | Nadler et al 2001 | Relationship between hip muscle imbalance and occurrence of low back pain in collegiate athletes: a prospective study. American Journal of Physical Medicine & Rehabilitation;80(8):572-7 | No separate data for LBP participants |
|  | Nadler et al 1998 | Low back pain in college athletes. A prospective study correlating lower extremity overuse or acquired ligamentous laxity with low back pain. Spine;23(7):828-33 | No separate data for LBP participants |
|  | Nelson-Wong et al 2014 | Transient low back pain development during standing predicts future clinical low back pain in previously asymptomatic individuals. Spine;39(6):E379-83 | No musculoskeletal risk factors assessed/reported |
|  | Nelson-Wong et al 2009 | Development of active hip abduction screening test for identifying occupational low back pain. Journal of Orthopaedic and Sports Physical therapy;39(9):649-57 | No follow up or follow up > 1 year |
|  | Newton et al 1993 | Trunk strength testing with iso-machines. Part 2: Experimental evaluation of the Cybex II Back Testing System in normal subjects and patients with chronic low back pain. Spine;18(7):812-24 | No follow up or follow up > 1 year |
|  | Ostenberg 2000 | Injury risk factors in female European football. A prospective study of 123 players during one season. Scandinavian Journal of Medicine & Science in Sports;10(5):279 | No separate data for LBP |
|  | Portus et al 2004 | Technique factors related to ball release speed and trunk injuries in high performance cricket fast bowlers. Sports Biomechanics;(32):263-84 | No musculoskeletal risk factors assessed/reported |
|  | Poussa et al 2005 | Anthropometric measurements and growth as predictors of low-back pain: A cohort study of children followed up from the age of 11 to 22 years. European Spine Journal;14(6):595-98 | No follow up or follow up > 1 year |
|  | Ramond-Roquin et al 2011 | Biomechanical constraints remain major risk factors for low back pain: Results from a prospective cohort study in french male employees. Occupational and Environmental Medicine;68:A98 | No musculoskeletal risk factors assessed/reported |
|  | Ramond-Roquin et al 2015 | Biomechanical constraints remain major risk factors for low back pain. Results from a prospective cohort study in French male employees. Spine;15(4):559-69 | No follow up or follow up > 1 year |
|  | Reynolds et al 2002 | Injury occurrence and risk factors in construction engineers and combat artillery soldiers. Military Medicine;167(12):971-7 | No separate data for LBP |
|  | Rissanen et al 2002 | Does good trunk extensor performance protect against back-related work disability? Journal of Rehabilitation Medicine;34(2):62-6 | No follow up or follow up > 1 year |
|  | Roussel et al 2009 | Altered lumbopelvic movement control but not generalized joint hypermobility is associated with increased injury in dancers. A prospective study. Manual Therapy;14(6):630-5 | No separate data for LBP |
|  | Salminen et al 1995 | Low back pain in the young. A prospective three-year follow-up study of subjects with and without low back pain. Spine;20(19):2101-8 | No follow up or follow up > 1 year |
|  | Salminen et al 1999 | Recurrent low back pain and early disc degeneration in the young. Spine;24(13):1316-21 | No follow up or follow up > 1 year |
|  | Schmidt et al 2014 | Prevalence of Low Back Pain in Adolescent Athletes – an Epidemiological Investigation. International Journal of Sports Medicine;35(8):684-89 | Not prospective |
|  | Secer et al 2011 | Nonspecific low back pain in a group of young adult men. Turkish neurosurgery;21(2):135-9 | No musculoskeletal risk factors assessed/reported |
|  | Sjolie et al 2001 | The significance of high lumbar mobility and low lumbar strength for current and future low back pain in adolescents. Spine;26(23):2629-36 | No follow up or follow up > 1 year |
|  | Sitthipornvorakul et al 2015 | The effect of daily walking steps on preventing neck and low back  pain in sedentary workers: a 1-year prospective cohort study. European Spine Journal;24(3):417-24 | No musculoskeletal risk factors assessed/reported |
|  | Stevenson et al 2001 | A longitudinal study of the development of low back pain in an industrial population. Spine;26(12):1370-7 | No follow up or follow up > 1 year |
|  | Svensson et al 2008 | Factors predicting dropout in student nursing assistants. Occupational Medicine (Oxford);58(8):527-33 | Not prospective |
|  | Symmons et al 1991 | A longitudinal study of back pain and radiological changes in the lumbar spines of middle aged women. I. Clinical findings. Annals of the Rhematic Diseases;50(3):158-61 | No follow up or follow up > 1 year |
|  | Szeto et al 2007 | Work-related musculoskeletal disorders in urban bus drivers of Hong Kong. Journal of Occupational Rehabilitation;17(2):181-98 | Not prospective |
|  | Taanila et al 2010 | Aetiology and risk factors of musculoskeletal disorders in physically active conscripts: a follow-up study in the Finnish Defence Forces. BMC Musculoskeletal Disorders;11:146 | No separate data for LBP |
|  | Taanila et al 2012 | Predictors of low back pain in physically active conscripts with special emphasis on muscular fitness. Spine Journal: Official Journal of the North American Spine Society;12(9):737-48 | No musculoskeletal risk factors assessed/reported |
|  | Takala et al 2000 | Do functional tests predict low back pain? Spine;25(16):2126-32 | Unable to determine LBP status in asymptomatic group at follow up |
|  | Teichtahl et al 2015 | Physical inactivity is associated with narrower  lumbar intervertebral discs, high fat content of paraspinal muscles and low back pain and  disability. Arthritis Research & Therapy;17:114 | Not prospective |
|  | Timpka et al 2013 | Muscle strength in adolescent men and future musculoskeletal pain: A cohort study with 17 years of follow-up. BMJ Open;3(5):1-8 | No follow up or follow up > 1 year |
|  | Tobias et al 2013 | Joint hypermobility is a risk factor for musculoskeletal pain during adolescence: findings of a prospective cohort study. Arthritis & Rheumatism;65(4):1107-15 | No follow up or follow up > 1 year |
|  | Tsai et al 2011 | Risk factors for illness absence due to musculoskeletal disorders in a 4-year prospective study of a petroleum-manufacturing population. Journal of Occupational & Environmental Medicine;53(4):434-40 | No separate data for LBP |
|  | Vandergrift et al 2012 | Physical and psychosocial ergonomic risk factors for low back pain in automobile manufacturing workers. Occupational and Environmental Medicine;69(1):29-34 | No musculoskeletal risk factors assessed/reported |
|  | Vanti et al2010 | Low back pain in adolescent gymnasts. Prevalence and risk factors. Scienza Riabilitativa;12(2):45-50 | Not prospective |
|  | Wen et al 1997 | Lower extremity alignment and risk of overuse injuries in runners. Medicine and Science in Sports and Exercise;29(10):1291-8 | No separate data for LBP |
|  | Wilkerson et al 2012 | Prediction of core and lower extremity strains and sprains in collegiate football players: a preliminary study. Journal of Athletic Training;47(3):264-72 | No separate data for LBP |
|  | Wilkerson et al 2015 | A refined prediction model for core and lower extremity sprains and strains among collegiate football players. Journal of Athletic Training;50(6):643-50 | No separate data for LBP |
