# Supplementary material for: Restriction in lateral bending range of motion, lumbar lordosis, and hamstring flexibility predicts the development of low back pain: a systematic review of prospective cohort studies
Source: BMC Musculoskelet Disord. 2017 May 5;18:179. doi: 10.1186/s12891-017-1534-0 (PMC5418732; doi:10.1186/s12891-017-1534-0)
Supplement: Supplementary file 3 — Fully annotated forest plots for non-significant meta-analyses. (DOCX 449 kb) [file 12891_2017_1534_MOESM3_ESM.docx]

**Additional file 3. Annotated forest plots for nonsignificant meta-analyses**


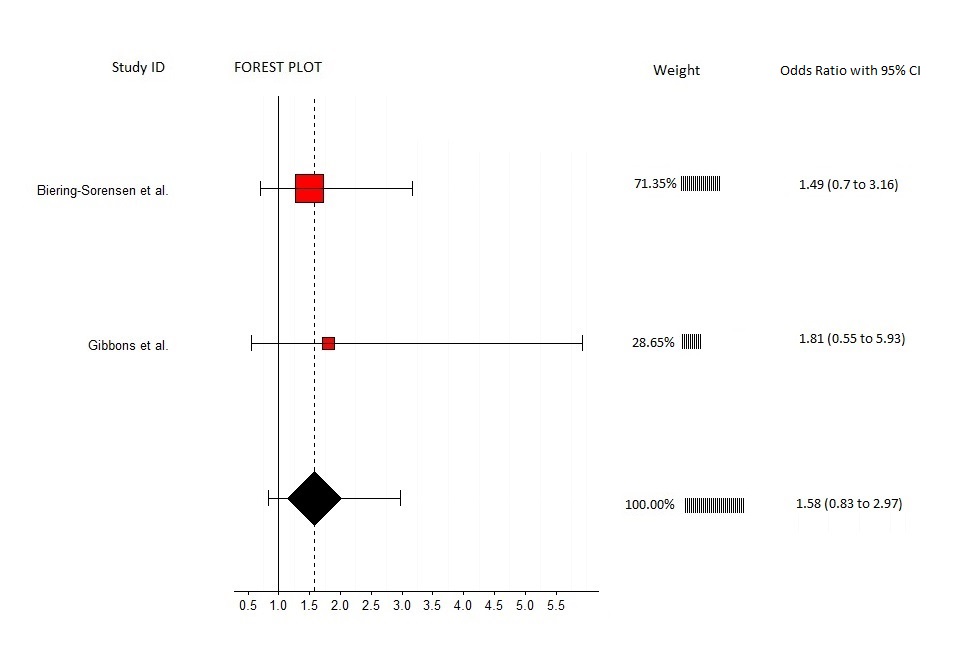


**Fully annotated forest plot for back muscle strength and LBP**


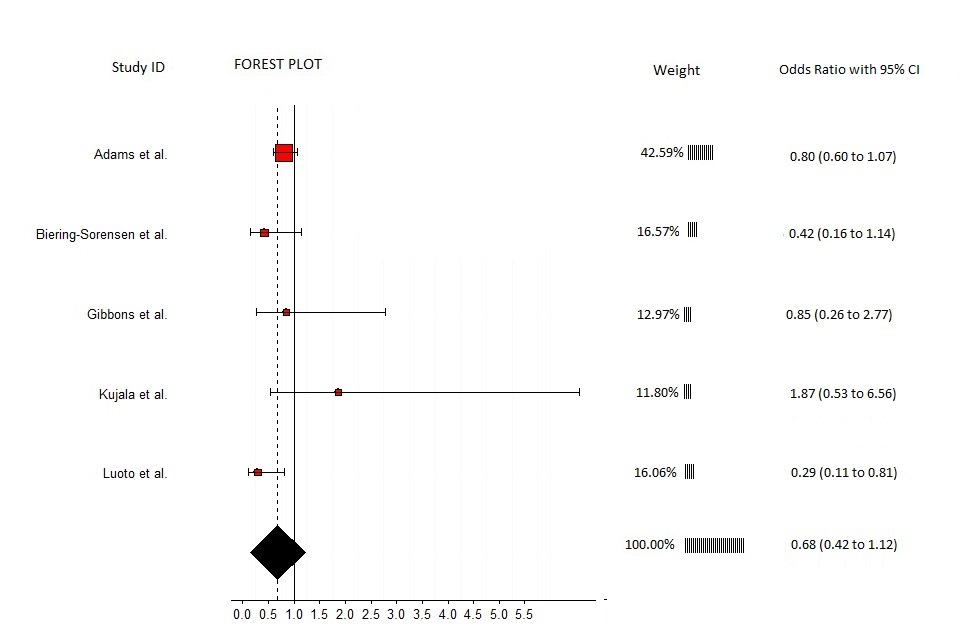


**Fully annotated forest plot for back muscle endurance and LBP**


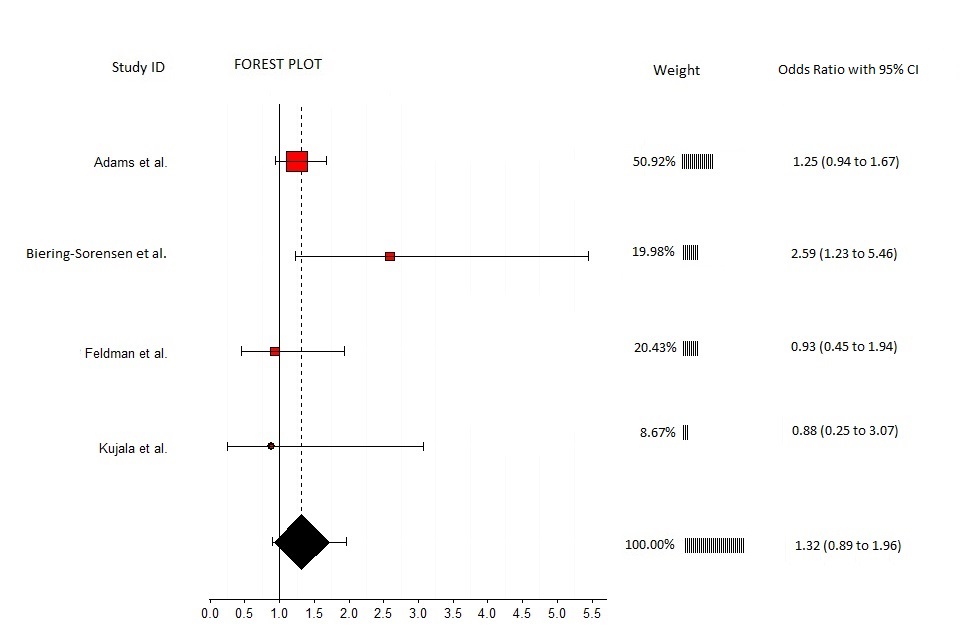


**Fully annotated forest plot for lumbar flexion range of motion and LBP**


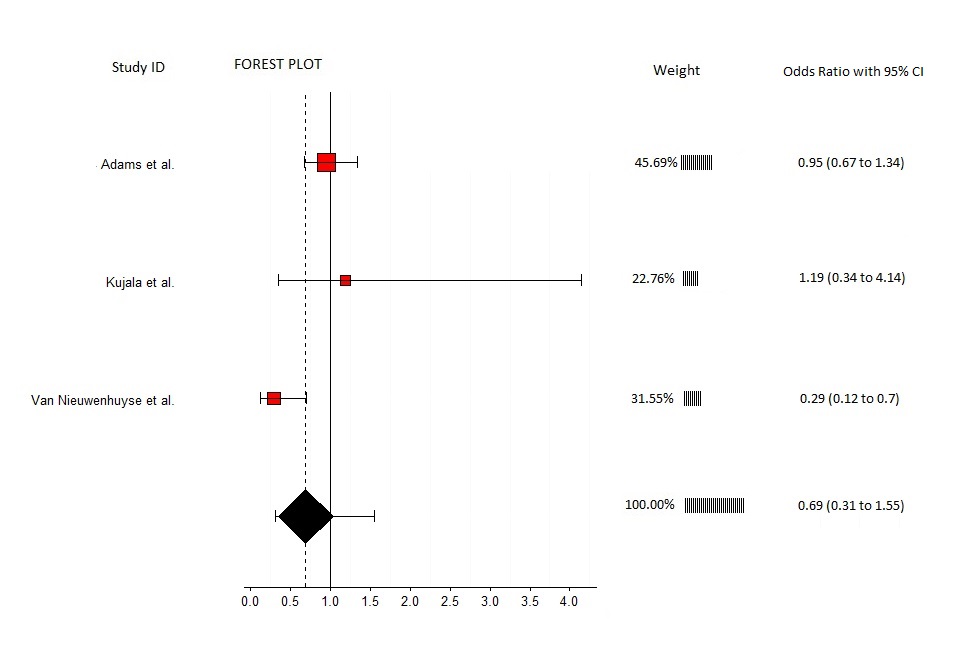


**Fully annotated forest plot for lumbar extension range of motion and LBP**


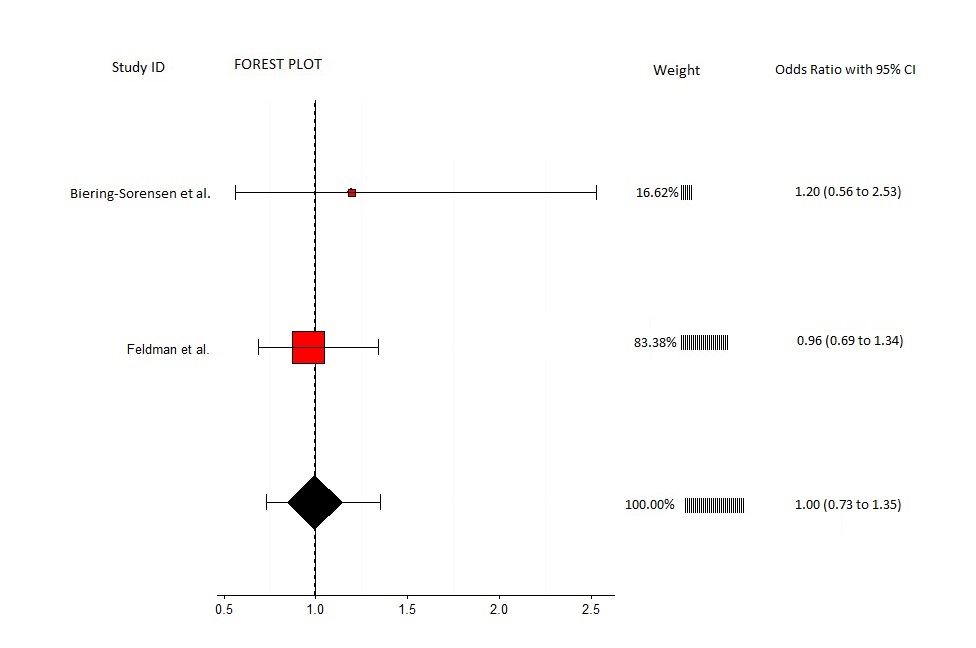


**Fully annotated forest plot for isometric abdominal strength and LBP**


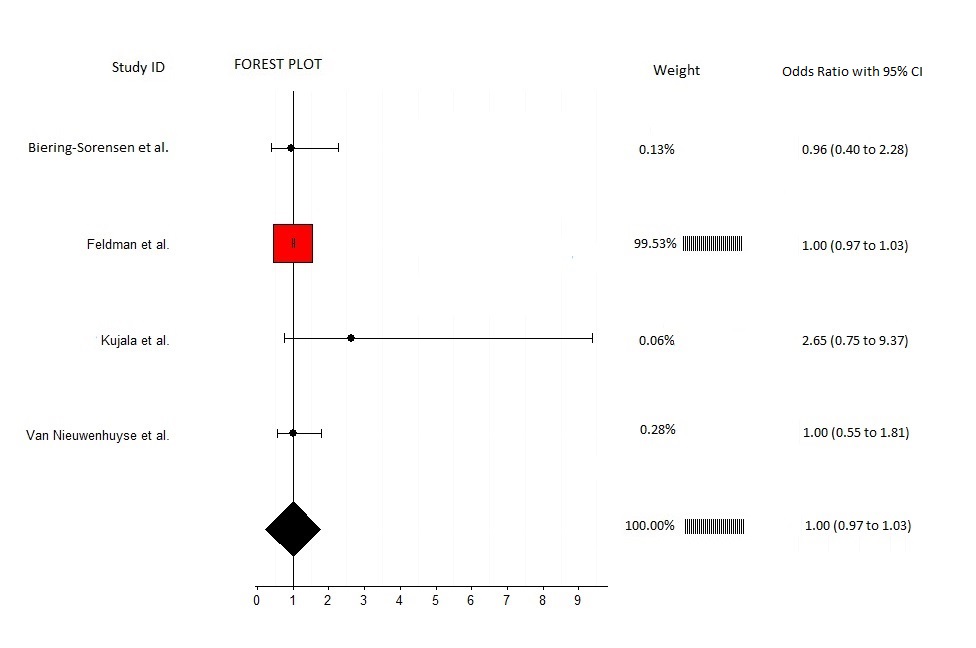


**Fully annotated forest plot for fingertip to floor distance and LBP**


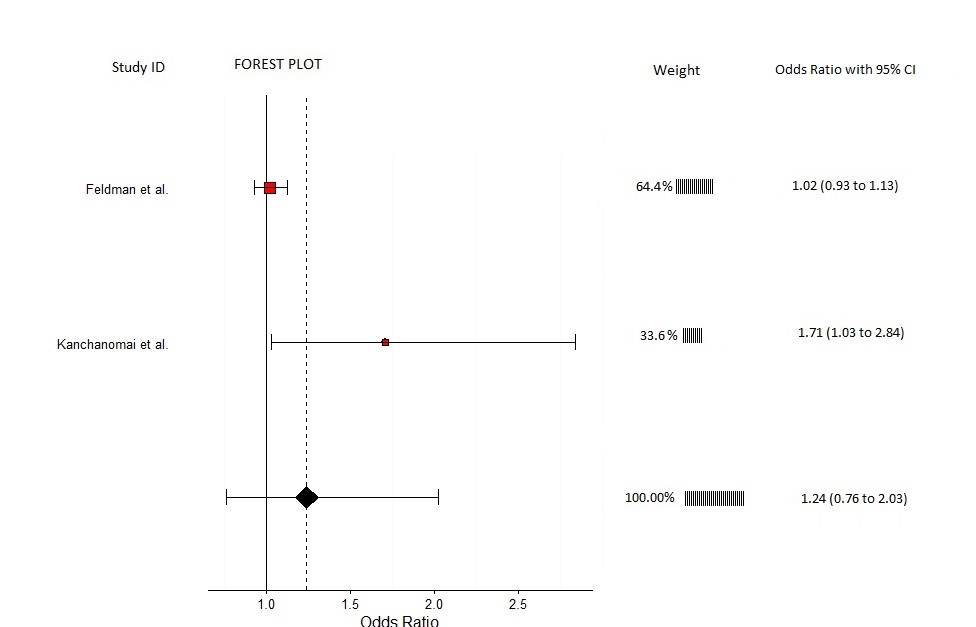


**Fully annotated forest plot for quadriceps flexibility and LBP**


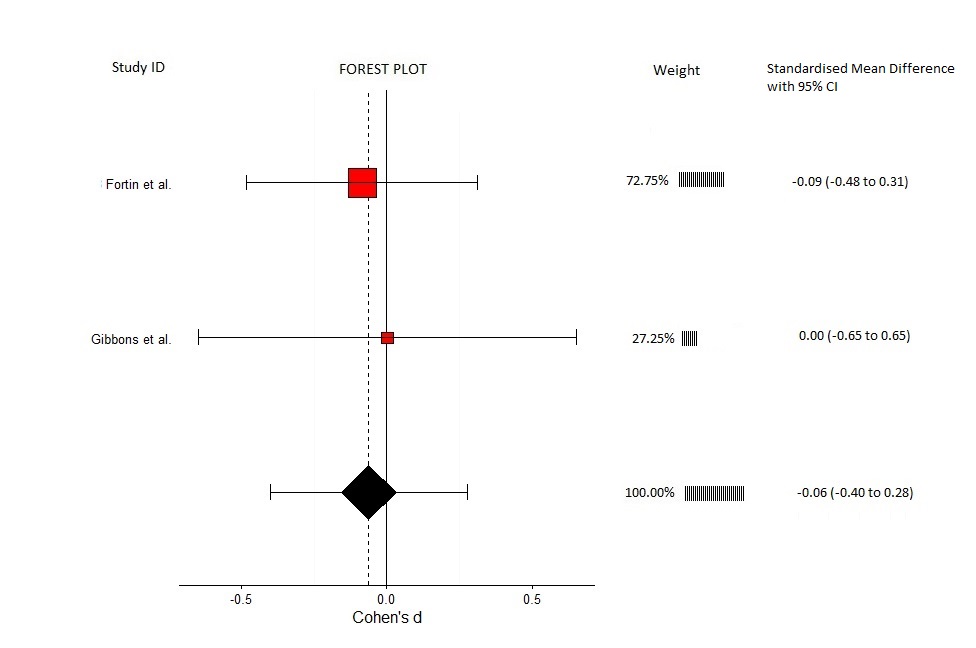


**Fully annotated forest plot for erector spinae cross sectional area and LBP**


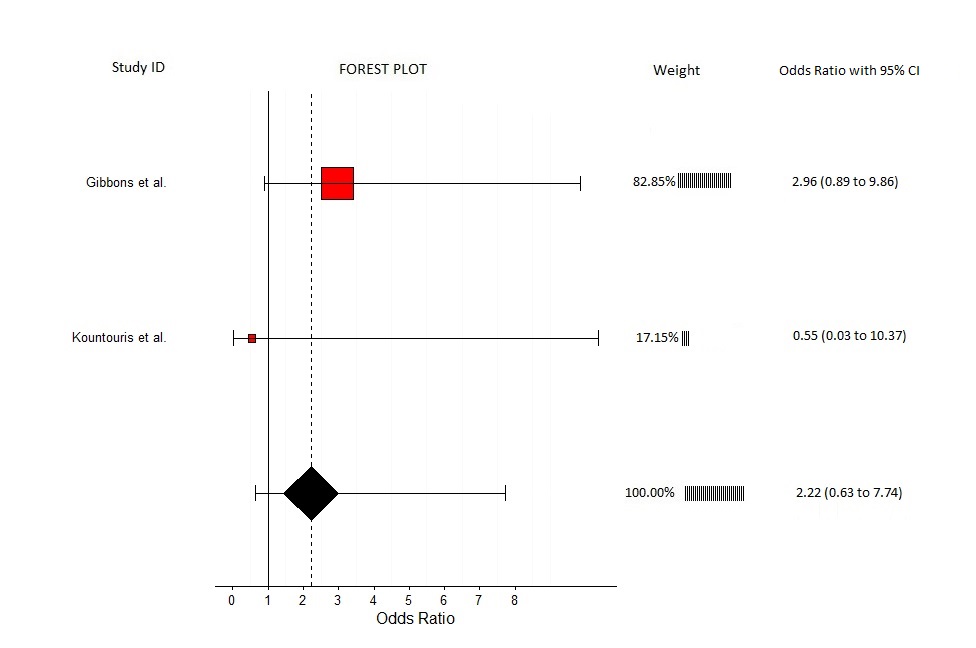


**Fully annotated forest plot for quadratus lumborum cross sectional area and LBP**
