# Supplementary material for: Restriction in lateral bending range of motion, lumbar lordosis, and hamstring flexibility predicts the development of low back pain: a systematic review of prospective cohort studies
Source: BMC Musculoskelet Disord. 2017 May 5;18:179. doi: 10.1186/s12891-017-1534-0 (PMC5418732; doi:10.1186/s12891-017-1534-0)
Supplement: Supplementary file 4 — Overview of risk factors measured in single studies. (DOCX 20 kb) [file 12891_2017_1534_MOESM4_ESM.docx]

**Additional file 4. Overview of risk factors in single studies**

| Study | Risk factor | Measurement technique | Result |
| --- | --- | --- | --- |
| Adams et al. (1999) [29] | Quadriceps strength | Equipment: Thornvall chair  Method: MVC in seated position with knee flexed at 90° | Not reported |
|  | Sacral inclination angle | Equipment: 3Space Isotrak device  Method: Sensor on sacrum measured relative to vertical in upright standing | Any LBP: *p* = 0.800 |
|  | Hip flexion ROM | Equipment: 3Space Isotrak device  Method: Sensor on sacrum measured relative to vertical in upright standing then same again with participants in max hip flexion | Any LBP: *p* = 0.197 |
|  | Hip extension ROM | Equipment: 3Space Isotrak device  Method: Sensor on sacrum measured relative to vertical in upright standing then same again with participants in max hip extension | Not reported |
|  | Peak spinal loading | Equipment: 3Space Isotrak device  Method: Not clear | Not reported |
| Biering-Sorensen et al. (1984) [30] | Ratio between max flexion and extension of trunk | Equipment: Strain gauge dynamometer  Methods: Device attached to shoulders of participant and the MVC of 3 attempts of flexion was divided by the MVC of 3 attempts of extension both in a standing position. | Not reported |
| Fortin et al. (2015) [40] | Multifidus CSA | Equipment: 1.5 Tesla Magnetom SP 4000 magnetic resonance imager  Method: T2-weighted techniques at L3-4 and L5-S1 | More frequent LBP vs no LBP at follow up, *p* = >0.05 |
|  | Multifidus FCSA/CSA | Equipment: 1.5 Tesla Magnetom SP 4000 magnetic resonance imager  Method: T2-weighted techniques at L3-4 and L5-S1 | More frequent LBP vs no LBP at follow up, *p* = >0.05 |
|  | Multifidus CSA asymmetry | Equipment: 1.5 Tesla Magnetom SP 4000 magnetic resonance imager  Method: T2-weighted techniques at L3-4 and L5-S1 | More frequent LBP vs no LBP at follow up, *p* = >0.05 |
|  | Multifidus FCSA/CSA side to side difference | Equipment: 1.5 Tesla Magnetom SP 4000 magnetic resonance imager  Method: T2-weighted techniques at L3-4 and L5-S1 | More frequent LBP vs no LBP at follow up, *p* = >0.05 |
|  | Erector spinae FCSA/CSA | Equipment: 1.5 Tesla Magnetom SP 4000 magnetic resonance imager  Method: T2-weighted techniques at L3-4 and L5-S1 | More frequent LBP vs no LBP at follow up, *p* = >0.05 |
|  | Erector spinae CSA asymmetry | Equipment: 1.5 Tesla Magnetom SP 4000 magnetic resonance imager  Method: T2-weighted techniques at L3-4 and L5-S1 | More frequent LBP vs no LBP at follow up, *p* = >0.05 |
|  | Erector spinae FCSA/CSA side to side difference | Equipment: 1.5 Tesla Magnetom SP 4000 magnetic resonance imager  Method: T2-weighted techniques at L3-4 and L5-S1 | More frequent LBP vs no LBP at follow up, *p* = >0.05 |
| Gibbons et al. (1997) [32] | Psychophysical lifting strength | Equipment: not clear  Methods: Participants were asked to use the strength they thought they could comfortable maintain for 5 seconds without straining. Performed in a forward flexion position with straight arms and legs. | LBP vs no LBP at follow up, *p* = 0.33 |
|  | Psoas major CSA | Equipment: 1.5 tesla Magnetom magnetic resonance imager  Method: Slice thickness was 3 mm and gaps between the slices 0.3 mm at the L3-4 level. | LBP vs no LBP at follow up, *p* = 0.48 |
|  | Total paraspinal CSA | Equipment: 1.5 tesla Magnetom magnetic resonance imager  Method: Slice thickness was 3 mm and gaps between the slices 0.3 mm at the L3-4 level. | LBP vs no LBP at follow up, *p* = 0.58 |
|  | Proton density-weight signal erector spinae | Equipment: 1.5 tesla Magnetom magnetic resonance imager  Method: Slice thickness was 3 mm and gaps between the slices 0.3 mm at the L3-4 level. | LBP vs no LBP at follow up, *p* = 0.58 |
|  | Proton density-weight signal quadratus lumborum | Equipment: 1.5 tesla Magnetom magnetic resonance imager  Method: Slice thickness was 3 mm and gaps between the slices 0.3 mm at the L3-4 level. | LBP vs no LBP at follow up, *p* = 0.70 |
|  | Proton density-weight signal psoas major | Equipment: 1.5 tesla Magnetom magnetic resonance imager  Method: Slice thickness was 3 mm and gaps between the slices 0.3 mm at the L3-4 level. | LBP vs no LBP at follow up, *p* = 0.83 |
|  | T2-weighted signal intensity erector spinae | Equipment: 1.5 tesla Magnetom magnetic resonance imager  Method: Slice thickness was 3 mm and gaps between the slices 0.3 mm at the L3-4 level. | LBP vs no LBP at follow up, *p* = 0.21 |
|  | T2-weighted signal intensity quadratus lumborum | Equipment: 1.5 tesla Magnetom magnetic resonance imager  Method: Slice thickness was 3 mm and gaps between the slices 0.3 mm at the L3-4 level. | LBP vs no LBP at follow up, *p* = 0.22 |
|  | T2-weighted signal intensity psoas major | Equipment: 1.5 tesla Magnetom magnetic resonance imager  Method: Slice thickness was 3 mm and gaps between the slices 0.3 mm at the L3-4 level. | LBP vs no LBP at follow up, *p* = 0.95 |
| Kanchanomai et al. (2015) [33] | Lumbar flexion range of motion | Equipment: Tape measure  Method: Modified Schober | Not reported |
|  | Lateral flexion range of motion | Equipment: Tape measure  Methods: Difference between middle finger position on ipsilateral thigh to most distal position of middle finger achieved in max lateral flexion | Not reported |
|  | Hamstring length | Equipment: Goniometer  Methods: Supine active knee extension test | Not reported |
|  | Trunk extensor strength | Equipment: Stopwatch  Methods: Biering-Sorensen test | Not reported |
|  | Trunk flexor strength | Equipment: Stopwatch  Methods: Ito test | Not reported |
| Kujala et al. (1994) [35] | Hip flexor tightness | Equipment: Hydrogoniometer  Method: Not clear | *p* = 0.04 (stepwise discriminant analysis adjusting for physical activity group in boys only) |
|  | Abdominal endurance | Equipment: Stopwatch  Method: Participants instructed to hold a sit-up for a max of 240 seconds | No significant differences |
|  | Hypermobility index | Equipment: None  Method: Beighton hypermobility assessment | No significant differences |
| Milgrom et al. (1993) [37] | Lumbar flexion range of motion | Equipment: Tape measure  Method: Modified Schober | Not reported |
|  | Quadriceps strength | Equipment: Modified Dan Lurie knee machine  Method: Max isometric quadriceps strength measured in 85° of knee flexion | Not reported |
|  | Hamstring strength | Equipment: Modified Dan Lurie knee machine  Method: Max isometric hamstring strength measured in 15° of knee flexion | Not reported |
|  | Ankle plantarflexor strength | Equipment: Not clear  Method: Max isometric ankle plantarflexor strength measured in 5° of plantarflexion | Not reported |
|  | Abdominal power | Equipment: Stopwatch  Method: Max number of bent knee sit-ups done in 1 minute | Not reported |
| Nissinen et al. (1994) [39] | Pelvic tilt | Equipment: Not clear  Method: Not clear | LBP (girls): univariate OR = 1.92 (95% CI = 0.77-4.80)  LBP (boys): univariate OR = 0.89 (95% CI = 0.35-2.23) |
|  | Lumbar hump size | Equipment: Tape measure  Method: Distance from horizontal to concave side of abdomen in max forward flexion | LBP: Multivariate OR (adjusted for all variables) =1.19 (95% CI = 1.00-1.39) |
| Van Nieuwenhuyse et al. (2009) [38] | Iliac crest inequity | Equipment: Not clear  Method: Not clear | >1.5cm iliac crest inequality for LBP: RR = 0.72 (95% CI 0.27-1.98, *p* = 0.673) |

MVC: max voluntary contraction; LBP: low back pain; CSA: cross sectional area; FCSA: functional cross sectional area; OR: odds ratio; 95% CI: 95% confidence interval; RR: relative risk
